# Supplementary material for: Pyruvate Dehydrogenase Kinase 1 inhibition mediated oxidative phosphorylation enhancement in cartilage promotes osteoarthritis progression
Source: BMC Musculoskelet Disord. 2023 Jul 20;24:597. doi: 10.1186/s12891-023-06585-6 (PMC10357736; doi:10.1186/s12891-023-06585-6)
Supplement: Supplementary file 1 — Additional file 1. [file 12891_2023_6585_MOESM1_ESM.zip › BP.Gsea.1653623667859/GOBP_NUCLEOSIDE_BISPHOSPHATE_METABOLIC_PROCESS.html]

Details for gene set GOBP\_NUCLEOSIDE\_BISPHOSPHATE\_METABOLIC\_PROCESS[GSEA]

|  || Dataset | 55457\_collapsed\_to\_symbols.cls.cls #OA\_versus\_normal.cls.cls #OA\_versus\_normal\_repos |
| Phenotype | cls.cls#OA\_versus\_normal\_repos |
| Upregulated in class | OA |
| GeneSet | GOBP\_NUCLEOSIDE\_BISPHOSPHATE\_METABOLIC\_PROCESS |
| Enrichment Score (ES) | 0.3054102 |
| Normalized Enrichment Score (NES) | 1.4247557 |
| Nominal p-value | 0.02406417 |
| FDR q-value | 0.46287265 |
| FWER p-Value | 1.0 |
Table: GSEA Results Summary

  

Fig 1: Enrichment plot: GOBP\_NUCLEOSIDE\_BISPHOSPHATE\_METABOLIC\_PROCESS      
 Profile of the Running ES Score & Positions of GeneSet Members on the Rank Ordered List

  

| SYMBOL | TITLE | RANK IN GENE LIST | RANK METRIC SCORE | RUNNING ES | CORE ENRICHMENT || 1 | MPC2 | mitochondrial pyruvate carrier 2 [Source:HGNC Symbol;Acc:HGNC:24515] | 140 | 0.870 | 0.0180 | Yes |
| 2 | PDHB | pyruvate dehydrogenase E1 subunit beta [Source:HGNC Symbol;Acc:HGNC:8808] | 149 | 0.862 | 0.0461 | Yes |
| 3 | PANK2 | pantothenate kinase 2 [Source:HGNC Symbol;Acc:HGNC:15894] | 220 | 0.805 | 0.0675 | Yes |
| 4 | PMVK | phosphomevalonate kinase [Source:HGNC Symbol;Acc:HGNC:9141] | 346 | 0.725 | 0.0819 | Yes |
| 5 | DLD | dihydrolipoamide dehydrogenase [Source:HGNC Symbol;Acc:HGNC:2898] | 565 | 0.635 | 0.0860 | Yes |
| 6 | PAPSS2 | 3'-phosphoadenosine 5'-phosphosulfate synthase 2 [Source:HGNC Symbol;Acc:HGNC:8604] | 592 | 0.627 | 0.1048 | Yes |
| 7 | PAPSS1 | 3'-phosphoadenosine 5'-phosphosulfate synthase 1 [Source:HGNC Symbol;Acc:HGNC:8603] | 703 | 0.593 | 0.1160 | Yes |
| 8 | PPCS | phosphopantothenoylcysteine synthetase [Source:HGNC Symbol;Acc:HGNC:25686] | 716 | 0.589 | 0.1347 | Yes |
| 9 | HSD17B12 | hydroxysteroid 17-beta dehydrogenase 12 [Source:HGNC Symbol;Acc:HGNC:18646] | 795 | 0.570 | 0.1476 | Yes |
| 10 | ACOT7 | acyl-CoA thioesterase 7 [Source:HGNC Symbol;Acc:HGNC:24157] | 971 | 0.524 | 0.1514 | Yes |
| 11 | ACAT1 | acetyl-CoA acetyltransferase 1 [Source:HGNC Symbol;Acc:HGNC:93] | 1017 | 0.513 | 0.1650 | Yes |
| 12 | PPT1 | palmitoyl-protein thioesterase 1 [Source:HGNC Symbol;Acc:HGNC:9325] | 1042 | 0.508 | 0.1801 | Yes |
| 13 | TECR | "trans-2,3-enoyl-CoA reductase [Source:HGNC Symbol;Acc:HGNC:4551]" | 1047 | 0.508 | 0.1967 | Yes |
| 14 | ACOT8 | acyl-CoA thioesterase 8 [Source:HGNC Symbol;Acc:HGNC:15919] | 1088 | 0.500 | 0.2102 | Yes |
| 15 | TPST2 | tyrosylprotein sulfotransferase 2 [Source:HGNC Symbol;Acc:HGNC:12021] | 1197 | 0.477 | 0.2177 | Yes |
| 16 | OXSM | "3-oxoacyl-ACP synthase, mitochondrial [Source:HGNC Symbol;Acc:HGNC:26063]" | 1342 | 0.451 | 0.2215 | Yes |
| 17 | HSD17B4 | hydroxysteroid 17-beta dehydrogenase 4 [Source:HGNC Symbol;Acc:HGNC:5213] | 1354 | 0.450 | 0.2356 | Yes |
| 18 | SUCLG2 | succinate-CoA ligase GDP-forming subunit beta [Source:HGNC Symbol;Acc:HGNC:11450] | 1472 | 0.432 | 0.2409 | Yes |
| 19 | PDK2 | pyruvate dehydrogenase kinase 2 [Source:HGNC Symbol;Acc:HGNC:8810] | 1772 | 0.388 | 0.2304 | Yes |
| 20 | TDO2 | "tryptophan 2,3-dioxygenase [Source:HGNC Symbol;Acc:HGNC:11708]" | 1783 | 0.387 | 0.2426 | Yes |
| 21 | SNCA | synuclein alpha [Source:HGNC Symbol;Acc:HGNC:11138] | 1800 | 0.384 | 0.2541 | Yes |
| 22 | ACSM3 | acyl-CoA synthetase medium chain family member 3 [Source:HGNC Symbol;Acc:HGNC:10522] | 1835 | 0.380 | 0.2641 | Yes |
| 23 | HMGCS2 | 3-hydroxy-3-methylglutaryl-CoA synthase 2 [Source:HGNC Symbol;Acc:HGNC:5008] | 1888 | 0.374 | 0.2725 | Yes |
| 24 | ELOVL4 | ELOVL fatty acid elongase 4 [Source:HGNC Symbol;Acc:HGNC:14415] | 2017 | 0.360 | 0.2745 | Yes |
| 25 | PDK1 | pyruvate dehydrogenase kinase 1 [Source:HGNC Symbol;Acc:HGNC:8809] | 2124 | 0.347 | 0.2778 | Yes |
| 26 | PPCDC | phosphopantothenoylcysteine decarboxylase [Source:HGNC Symbol;Acc:HGNC:28107] | 2251 | 0.332 | 0.2790 | Yes |
| 27 | SULT1A1 | sulfotransferase family 1A member 1 [Source:HGNC Symbol;Acc:HGNC:11453] | 2410 | 0.313 | 0.2771 | Yes |
| 28 | PDHA1 | pyruvate dehydrogenase E1 subunit alpha 1 [Source:HGNC Symbol;Acc:HGNC:8806] | 2433 | 0.310 | 0.2857 | Yes |
| 29 | TPST1 | tyrosylprotein sulfotransferase 1 [Source:HGNC Symbol;Acc:HGNC:12020] | 2512 | 0.302 | 0.2897 | Yes |
| 30 | ACSBG1 | acyl-CoA synthetase bubblegum family member 1 [Source:HGNC Symbol;Acc:HGNC:29567] | 2520 | 0.301 | 0.2992 | Yes |
| 31 | CROT | carnitine O-octanoyltransferase [Source:HGNC Symbol;Acc:HGNC:2366] | 2567 | 0.295 | 0.3054 | Yes |
| 32 | SULT1E1 | sulfotransferase family 1E member 1 [Source:HGNC Symbol;Acc:HGNC:11377] | 2791 | 0.271 | 0.2970 | No |
| 33 | SULT1A2 | sulfotransferase family 1A member 2 [Source:HGNC Symbol;Acc:HGNC:11454] | 2881 | 0.262 | 0.2988 | No |
| 34 | FAR2 | fatty acyl-CoA reductase 2 [Source:HGNC Symbol;Acc:HGNC:25531] | 2999 | 0.250 | 0.2980 | No |
| 35 | HMGCR | 3-hydroxy-3-methylglutaryl-CoA reductase [Source:HGNC Symbol;Acc:HGNC:5006] | 3047 | 0.245 | 0.3025 | No |
| 36 | SUCLA2 | succinate-CoA ligase ADP-forming subunit beta [Source:HGNC Symbol;Acc:HGNC:11448] | 3228 | 0.228 | 0.2960 | No |
| 37 | ELOVL6 | ELOVL fatty acid elongase 6 [Source:HGNC Symbol;Acc:HGNC:15829] | 3253 | 0.226 | 0.3017 | No |
| 38 | ENPP1 | ectonucleotide pyrophosphatase/phosphodiesterase 1 [Source:HGNC Symbol;Acc:HGNC:3356] | 3392 | 0.212 | 0.2980 | No |
| 39 | COASY | Coenzyme A synthase [Source:HGNC Symbol;Acc:HGNC:29932] | 3498 | 0.202 | 0.2965 | No |
| 40 | GLYAT | glycine-N-acyltransferase [Source:HGNC Symbol;Acc:HGNC:13734] | 3871 | 0.167 | 0.2730 | No |
| 41 | SULT1A3 | sulfotransferase family 1A member 3 [Source:HGNC Symbol;Acc:HGNC:11455] | 3923 | 0.162 | 0.2745 | No |
| 42 | DGAT1 | diacylglycerol O-acyltransferase 1 [Source:HGNC Symbol;Acc:HGNC:2843] | 3950 | 0.160 | 0.2778 | No |
| 43 | ACOT2 | acyl-CoA thioesterase 2 [Source:HGNC Symbol;Acc:HGNC:18431] | 4046 | 0.152 | 0.2754 | No |
| 44 | ACSM2A | acyl-CoA synthetase medium chain family member 2A [Source:HGNC Symbol;Acc:HGNC:32017] | 4080 | 0.148 | 0.2778 | No |
| 45 | MCCC2 | methylcrotonyl-CoA carboxylase subunit 2 [Source:HGNC Symbol;Acc:HGNC:6937] | 4363 | 0.123 | 0.2599 | No |
| 46 | MLYCD | malonyl-CoA decarboxylase [Source:HGNC Symbol;Acc:HGNC:7150] | 4852 | 0.083 | 0.2246 | No |
| 47 | ACSM5 | acyl-CoA synthetase medium chain family member 5 [Source:HGNC Symbol;Acc:HGNC:26060] | 4976 | 0.074 | 0.2174 | No |
| 48 | HACD1 | 3-hydroxyacyl-CoA dehydratase 1 [Source:HGNC Symbol;Acc:HGNC:9639] | 5342 | 0.044 | 0.1904 | No |
| 49 | PANK4 | pantothenate kinase 4 (inactive) [Source:HGNC Symbol;Acc:HGNC:19366] | 5382 | 0.041 | 0.1887 | No |
| 50 | ACOT9 | acyl-CoA thioesterase 9 [Source:HGNC Symbol;Acc:HGNC:17152] | 5451 | 0.037 | 0.1847 | No |
| 51 | DLAT | dihydrolipoamide S-acetyltransferase [Source:HGNC Symbol;Acc:HGNC:2896] | 5647 | 0.020 | 0.1701 | No |
| 52 | ACSBG2 | acyl-CoA synthetase bubblegum family member 2 [Source:HGNC Symbol;Acc:HGNC:24174] | 5970 | -0.002 | 0.1451 | No |
| 53 | HACD2 | 3-hydroxyacyl-CoA dehydratase 2 [Source:HGNC Symbol;Acc:HGNC:9640] | 6162 | -0.017 | 0.1307 | No |
| 54 | ACSL6 | acyl-CoA synthetase long chain family member 6 [Source:HGNC Symbol;Acc:HGNC:16496] | 6505 | -0.043 | 0.1054 | No |
| 55 | SLC25A1 | solute carrier family 25 member 1 [Source:HGNC Symbol;Acc:HGNC:10979] | 6509 | -0.043 | 0.1066 | No |
| 56 | PDHX | pyruvate dehydrogenase complex component X [Source:HGNC Symbol;Acc:HGNC:21350] | 6760 | -0.060 | 0.0891 | No |
| 57 | PIPOX | pipecolic acid and sarcosine oxidase [Source:HGNC Symbol;Acc:HGNC:17804] | 6781 | -0.061 | 0.0896 | No |
| 58 | SLC26A1 | solute carrier family 26 member 1 [Source:HGNC Symbol;Acc:HGNC:10993] | 7618 | -0.128 | 0.0286 | No |
| 59 | PDK3 | pyruvate dehydrogenase kinase 3 [Source:HGNC Symbol;Acc:HGNC:8811] | 7661 | -0.132 | 0.0297 | No |
| 60 | ACOT11 | acyl-CoA thioesterase 11 [Source:HGNC Symbol;Acc:HGNC:18156] | 7746 | -0.137 | 0.0277 | No |
| 61 | ELOVL2 | ELOVL fatty acid elongase 2 [Source:HGNC Symbol;Acc:HGNC:14416] | 8080 | -0.161 | 0.0071 | No |
| 62 | ACSL3 | acyl-CoA synthetase long chain family member 3 [Source:HGNC Symbol;Acc:HGNC:3570] | 8170 | -0.169 | 0.0058 | No |
| 63 | ACLY | ATP citrate lyase [Source:HGNC Symbol;Acc:HGNC:115] | 8173 | -0.169 | 0.0112 | No |
| 64 | GCDH | glutaryl-CoA dehydrogenase [Source:HGNC Symbol;Acc:HGNC:4189] | 8180 | -0.170 | 0.0164 | No |
| 65 | ACSM1 | acyl-CoA synthetase medium chain family member 1 [Source:HGNC Symbol;Acc:HGNC:18049] | 8210 | -0.172 | 0.0199 | No |
| 66 | ELOVL1 | ELOVL fatty acid elongase 1 [Source:HGNC Symbol;Acc:HGNC:14418] | 8406 | -0.186 | 0.0109 | No |
| 67 | OGDH | oxoglutarate dehydrogenase [Source:HGNC Symbol;Acc:HGNC:8124] | 8503 | -0.194 | 0.0098 | No |
| 68 | ABCD1 | ATP binding cassette subfamily D member 1 [Source:HGNC Symbol;Acc:HGNC:61] | 8930 | -0.226 | -0.0159 | No |
| 69 | SULT2A1 | sulfotransferase family 2A member 1 [Source:HGNC Symbol;Acc:HGNC:11458] | 8934 | -0.227 | -0.0086 | No |
| 70 | PDHA2 | pyruvate dehydrogenase E1 subunit alpha 2 [Source:HGNC Symbol;Acc:HGNC:8807] | 9124 | -0.241 | -0.0153 | No |
| 71 | ACSL5 | acyl-CoA synthetase long chain family member 5 [Source:HGNC Symbol;Acc:HGNC:16526] | 9136 | -0.243 | -0.0081 | No |
| 72 | ACACA | acetyl-CoA carboxylase alpha [Source:HGNC Symbol;Acc:HGNC:84] | 9177 | -0.246 | -0.0030 | No |
| 73 | ACSF2 | acyl-CoA synthetase family member 2 [Source:HGNC Symbol;Acc:HGNC:26101] | 9219 | -0.250 | 0.0021 | No |
| 74 | PANK3 | pantothenate kinase 3 [Source:HGNC Symbol;Acc:HGNC:19365] | 9286 | -0.255 | 0.0055 | No |
| 75 | SLC26A2 | solute carrier family 26 member 2 [Source:HGNC Symbol;Acc:HGNC:10994] | 9523 | -0.276 | -0.0038 | No |
| 76 | SULT2B1 | sulfotransferase family 2B member 1 [Source:HGNC Symbol;Acc:HGNC:11459] | 9696 | -0.291 | -0.0075 | No |
| 77 | PPT2 | palmitoyl-protein thioesterase 2 [Source:HGNC Symbol;Acc:HGNC:9326] | 10103 | -0.331 | -0.0282 | No |
| 78 | BAAT | bile acid-CoA:amino acid N-acyltransferase [Source:HGNC Symbol;Acc:HGNC:932] | 10523 | -0.376 | -0.0484 | No |
| 79 | DLST | dihydrolipoamide S-succinyltransferase [Source:HGNC Symbol;Acc:HGNC:2911] | 10634 | -0.386 | -0.0441 | No |
| 80 | ACSL1 | acyl-CoA synthetase long chain family member 1 [Source:HGNC Symbol;Acc:HGNC:3569] | 10705 | -0.394 | -0.0364 | No |
| 81 | HMGCS1 | 3-hydroxy-3-methylglutaryl-CoA synthase 1 [Source:HGNC Symbol;Acc:HGNC:5007] | 10829 | -0.408 | -0.0324 | No |
| 82 | MVK | mevalonate kinase [Source:HGNC Symbol;Acc:HGNC:7530] | 10867 | -0.412 | -0.0216 | No |
| 83 | SULT1B1 | sulfotransferase family 1B member 1 [Source:HGNC Symbol;Acc:HGNC:17845] | 10893 | -0.416 | -0.0097 | No |
| 84 | MVD | mevalonate diphosphate decarboxylase [Source:HGNC Symbol;Acc:HGNC:7529] | 10934 | -0.423 | 0.0013 | No |
| 85 | ACACB | acetyl-CoA carboxylase beta [Source:HGNC Symbol;Acc:HGNC:85] | 10955 | -0.425 | 0.0139 | No |
| 86 | ELOVL5 | ELOVL fatty acid elongase 5 [Source:HGNC Symbol;Acc:HGNC:21308] | 11272 | -0.467 | 0.0048 | No |
| 87 | ACSL4 | acyl-CoA synthetase long chain family member 4 [Source:HGNC Symbol;Acc:HGNC:3571] | 11327 | -0.475 | 0.0164 | No |
| 88 | PDK4 | pyruvate dehydrogenase kinase 4 [Source:HGNC Symbol;Acc:HGNC:8812] | 11384 | -0.483 | 0.0281 | No |
| 89 | SLC25A16 | solute carrier family 25 member 16 [Source:HGNC Symbol;Acc:HGNC:10986] | 11411 | -0.488 | 0.0423 | No |
| 90 | DCAKD | dephospho-CoA kinase domain containing [Source:HGNC Symbol;Acc:HGNC:26238] | 11650 | -0.525 | 0.0412 | No |
| 91 | DIP2A | disco interacting protein 2 homolog A [Source:HGNC Symbol;Acc:HGNC:17217] | 12433 | -0.719 | 0.0041 | No |
| 92 | AASS | aminoadipate-semialdehyde synthase [Source:HGNC Symbol;Acc:HGNC:17366] | 12799 | -0.968 | 0.0079 | No |
Table: GSEA details [plain text format]

  

Fig 2: GOBP\_NUCLEOSIDE\_BISPHOSPHATE\_METABOLIC\_PROCESS      
 Blue-Pink O' Gram in the Space of the Analyzed GeneSet

  

Fig 3: GOBP\_NUCLEOSIDE\_BISPHOSPHATE\_METABOLIC\_PROCESS: Random ES distribution      
 Gene set null distribution of ES for **GOBP\_NUCLEOSIDE\_BISPHOSPHATE\_METABOLIC\_PROCESS**
